# Intolerance of Uncertainty and Attitudes towards Vaccination Impact Vaccinal Decision While Perceived Uncertainty Does Not

**DOI:** 10.3390/vaccines10101742

**Published:** 2022-10-18

**Authors:** Clémence Brun, Alexis Akinyemi, Laurène Houtin, Oulmann Zerhouni, Richard Monvoisin, Nicolas Pinsault

**Affiliations:** 1 AD-HOC Lab, 13 rue Hispano Suiza, 92270 Bois-Colombes, France; 2 TIMC-IMAG UMR CNRS 5525, ThEMAS Team, Université Grenoble Alpes, Domaine de la Merci, 5 Avenue du Grand Sablon, 38700 La Tronche, France; 3 Laboratoire Parisien de Psychologie Sociale, EA 4386 (équipe PS2C), 200 Av. de la République, CEDEX, 92001 Nanterre, France

**Keywords:** intolerance of uncertainty, uncertainty, decision-making, confidence, attitudes toward vaccination

## Abstract

The COVID-19 pandemic illustrated that intolerance of uncertainty (IU) can hinder problem-solving and lead to avoidance of ambiguous situations. Furthermore, people tend to lack confidence in decisions made in ambiguous contexts. We wanted to investigate the impact of IU on intentions to get vaccinated, to vaccinate one’s children, and to recommend the vaccine in situations with varying degrees of perceived uncertainty. We first conducted a pretest to select six scenarios with different levels of perceived uncertainty. In the core study, 485 participants answered for each of the six scenarios whether they would get vaccinated, vaccinate their children (or imagine doing so, for individuals without children), and whether they would recommend the vaccine. They also completed the IUS-12 (Intolerance of Uncertainty scale) and the VAX (Vaccination Attitudes Examination). Results showed that perceived uncertainty did not influence our measures, but the IUS-12 and VAX predicted the difference in score between the most and least uncertain scenarios. An indirect effect of the IUS-12 on decision confidence through the VAX was found, but with no direct effect. We conclude that, even if future studies should refine these results, Public Policies should be more focused on factors such as IU and attitudes toward vaccination.

## 1. Introduction

### 1.1. Decision-Making under Uncertainty

Everyday life is a succession of decisions made without necessarily being aware of the process by which we make them or the parameters that influence this process. Decision-making can be broadly defined as “the process of making choices, especially important choices “ [1], or as “the complex cognitive process of choosing an action from among several alternatives. The final choice results from this process, and this choice can be an opinion or an action” [2]. Many decisions are made in uncertain contexts, that is, a situation where information about the outcome of behavior remains unclear or limited [3,4,5,6]. 

Uncertainty can have a definite effect on decision-making, especially in relation to risk aversion (i.e., the preference for lower value rewards over more valuable but uncertain ones; [6,7,8]). Uncertainty management could therefore influence decision-making, as better management would minimize the impact of uncertainty on the decisions taken [9].

### 1.2. Impact of Intolerance of Uncertainty on Decision-Making

Intolerance of uncertainty (IU) is a variable of primary interest when investigating the impact of uncertainty on decision-making [4]. IU can be defined as “the tendency of an individual to consider as unacceptable the possibility that a negative event will occur, regardless of the probability of its occurrence” [10,11], as well as “the set of negative and positive psychological responses-cognitive; emotional; and behavioural-provoked by the conscious awareness of ignorance about particular aspects of the world” [12]. Authors such as Dugas and colleagues reported that IU can have deleterious effects on problem-solving abilities, which can ultimately lead to inaction and avoidance of situations perceived as ambiguous [10]. The same would apply to pure decision-making contexts, as suggested by Kornilova and colleagues’ study with their study using the Iowa Gambling Task [13]. Their results indicate that the IU modulates decision-making strategies under uncertainty. Other researchers, such as Luhmann and colleagues [14], have shown that a high IU is associated with giving up high monetary gains, if it means avoiding waiting in a state of uncertainty, in favor of smaller but immediately available gains. Waiting, especially in a state of uncertainty, could therefore influence decision-making and, consequently, behaviors.

### 1.3. Links between Confidence in One’s Own Judgement and Decision-Making

Confidence in one’s own judgment is another parameter of interest that stands out when looking at the literature on decision-making. Confidence can be defined as “a belief about the validity of our own thoughts, knowledge or performance and relies on a subjective feeling” [15]. A great deal of research has focused on the calibration between confidence in one’s decision and the quality of one’s decision, especially in medicine [16,17,18]. Decision accuracy and confidence are generally highly correlated, so that decisions in which all evidence points are chosen almost systematically and with have high confidence [19]. However, when evidence is ambiguous, confidence will be low, and individuals will try to remedy this situation by seeking out more information [19]. Nevertheless, seeking more information is often not possible and individuals can find themselves in a position of making choices without being fully confident in their decisions [20]. 

### 1.4. Pandemic Cases as Relevant Situations to Study Decision-Making

The COVID-19 pandemic is a perfect example of a situation in which one had to make a decision without all the information one would like to have, as individuals felt that there was insufficient information available to make a decision about getting vaccinated [21,22,23]. Using the unclear flow of information, individuals had to choose whether or not to get vaccinated and parents had to choose whether or not to vaccinate their children. This additional decision was further complicated by the fact that the cost-benefit balance of vaccination in children was highly controversial for COVID-19 [24]. Even if parents seemed to accept the vaccine (e.g., 72.6% acceptability of a free vaccine against COVID among parents in a sample of Chinese factory employees in September 2020; [25]), this did not necessarily result in their having intentions to vaccinate their children (e.g., the likelihood of child COVID-19 vaccination was as follows in March 2021 among US parents: very likely (28%), somewhat likely (18%), somewhat unlikely (9%), very unlikely (33%), and unsure (12%) [26]). However, the overall acceptance of the COVID-19 vaccine was associated with intentions to recommend the vaccine to family and friends [27].

The COVID-19 case illustrates that pandemic contexts are particularly conducive to the study of the impact of uncertainty on decision-making. This type of situation also makes it possible to study decision-making in relation to traits of interest, such as attitude towards vaccines, which, in the case of COVID-19, reduced the probability of people with negative attitudes towards vaccines being vaccinated [28], or IU, as previously stated (especially as the lack of studies linking IU and COVID-19 has already been identified [4]).

### 1.5. Aim of Our Study

The impact of uncertainty intolerance on decision-making processes has already been studied using a scenario methodology by Jensen and colleagues [29]. Their study showed that individuals highly intolerant of uncertainty became less confident in their decisions across various decision-making blocks and were less likely to change their decision if presented with more information about the presented situation and an alternative response option. However, as they did not control for the perceived level of uncertainty in their scenarios, they did not study the impact of the perceived level of uncertainty on the decisions taken. We wanted to address these limitations while incorporating the variables of interest that we identified in our study.

## 2. Pretest

### 2.1. Method

Inspired by Jensen et al.’s [29] methodology, we wanted to investigate the impact of IU on decision-making processes in a relevant context to the current global health pandemic and with a methodology that differentiates the observed effects according to different levels of perceived uncertainty. In addition, we wanted to include the literature covering the links between uncertainty and confidence in one’s own judgment and between uncertainty and attitudes toward vaccines by integrating these variables into our study. Based on the literature, we hypothesized that a higher level of IU will lead to lower intentions to get vaccinated, to have their children vaccinated, and to recommend the vaccine (H1); that a high level of negative attitudes towards vaccination would be associated with low intentions to get vaccinated, to have their children vaccinated and to recommend the vaccine (H2); and that a high level of IU would be associated with low levels of confidence in one’s decision (H3). 

#### 2.1.1. Population

The pretest was presented to a convenience sample recruited via social networks (e.g., Twitter) and via our own personal networks (e.g., email). We conducted the pretest on 950 French-speaking individuals. We excluded the data of 134 participants that did not meet our inclusion criteria (i.e., 11 participants because they were minors, 121 participants because they completed the entire questionnaire in less than one minute which is too fast for careful completion, and 2 participants who had a completion rate too low). The final sample consisted of 816 individuals (*M*_age_ = 27.55, *ET*_age_ = 11.76, 361 women, 152 men, 5 others, and 432 who did not specify their gender).

#### 2.1.2. Material

We used the Qualtrics survey design and distribution software (version: 05-2022, Qualtrics, Provo, UT, USA) to present several scenarios to the participants. We decided to present scenarios describing a pandemic context. This type of context seemed appropriate for our study insofar as this type of context can involve multiple forms of uncertainty (e.g., uncertainties related to the disease, its mode of transmission, its effects, the way to protect against it, the effects of potential treatments, etc.). We wrote 32 scenarios that implied differing levels of uncertainty. Specifically, these identical scenarios varied on five parameters modulating the level of uncertainty expressed in the scenario, with two modalities each (i.e., one modality indicating a high degree of uncertainty and the other a low degree of uncertainty). Details of the parameters and their modalities can be found in Table 1. An example of a parameter was “Knowledge about the virus”, for which the “High uncertainty” modality was “Recently discovered and not well known” and the “Low uncertainty” modality was “That we have known and studied for many years”. Thus, a scenario could have one, two, three, or four parameters with a “High uncertainty” modality. We decided to conduct a pretest to evaluate the average level of uncertainty felt by the participants when reading these scenarios. We aimed at classifying them and selecting six scenarios for the main phase of the study in which we presented scenarios evoking very different levels of uncertainty. Among the 32 scenarios we wrote, 2 were extracted to serve as reference scenarios for the participants (cf. Procedure). Pretesting was, therefore, carried out on the remaining 30 scenarios, with each participant reading 10 of them randomly chosen.

The survey concluded with socio-demographic questions. First, we asked participants’ gender. Response options were “male”, “female”, and “other”. If participants chose “other”, a blank space allowed them to fill in their gender. Age was assessed through the following open-ended question: “Please indicate your age in the box below (in numbers, e.g., 32)”.

#### 2.1.3. Procedure

The study began with a page introducing the study and giving instructions. After thanking the participants for their interest, we introduced the study as an investigation of the parameters influencing decision-making. The time required for completion was specified and the anonymity of the participants was assured. To ensure that we were measuring responses to a pandemic context but not necessarily to COVID-19, we then presented participants with a disclaimer stating that our study was not related to the pandemic (i.e., “You will read fictitious scenarios and then we will ask you to answer some questions. Please be aware that this study is not about opinions about the COVID vaccine, we are interested in your decision-making processes. Please give your opinion while trying to keep the current health context out of it”).

Afterward, each participant read 10 scenarios. We chose to extract from our list of 32 scenarios the 2 scenarios with the most extreme modalities for the five parameters to show them as examples to the participants. We aimed at giving them a better idea of what we meant when talking about uncertainty in a scenario. We chose to consider these two scenarios as being at the extreme ends of the continuum from “Low uncertainty” to “High uncertainty” and not to pretest them. These two scenarios, then considered reference scenarios, were presented to the participants in the following manner: 

##### For Reference, This Is an Extremely Certain Scenario

C2PO is a highly contagious virus that has been known and studied for many years. It mainly affects children and gives quite severe symptoms but we know that they will not leave any after-effects. The mode of transmission of the virus is well known. A vaccine has just been developed to fight against this pandemic which is getting worse. Its effectiveness rate is known with certainty and we are well aware of the possible side effects and their frequency.

##### This Is an Extremely Uncertain Scenario

C2PO is a recently discovered, highly contagious virus that is not well known. It mainly affects children and gives quite severe symptoms whose long-term consequences are unknown. The mode of transmission of the virus remains unknown. A vaccine has just been developed to fight against this pandemic which is getting worse. We have only partial data on its effectiveness and we do not yet know the risks that it will have side effects.

Of the remaining 30 scenarios, 10 of them were randomly selected and presented to each participant. We assigned a number to each scenario. All the scenarios are presented in Table 2 and the number of parameters in the “High uncertainty” modality is reported in Table 3. 

For each scenario, participants were asked to rate on an analogous scale from 0 to 100 how uncertain the context described in the scenario was to them (i.e., “How certain or uncertain do you think the health context described in the text you just read is? Use the slider below to answer (0 = extremely certain and 100 = extremely uncertain)”). After each reading and evaluation of a scenario, the participant moved on to the next until they had evaluated all 10 scenarios selected for them. Then, the study ended with socio-demographic questions (i.e., gender, age) and a thank you debriefing with an email address to contact with any comments or questions (i.e., “Your answers will help us to better understand how individuals make decisions. Please feel free to share this study with others, it would help us greatly! If you have any questions or comments, please do not hesitate to contact us at the following address: clemence.brun@adhoclab.fr). 

### 2.2. Results

#### 2.2.1. Analysis Process

We performed all our analyses using the R software (version: 2022.07.1, Joseph J. Allaire). We created four subgroups of scenarios according to the number of parameters in the “High uncertainty” modality. We aimed to select one scenario per subgroup, for a total of four selected scenarios. Of these four scenarios, we decided that the scenario with the lowest perceived uncertainty score and the one with the highest perceived uncertainty score would be necessarily included. For the selection of the other two scenarios, we decided to calculate the deciles and chose to keep the two scenarios with the perceived uncertainty score closest to the 30% and 70% order deciles. 

We, therefore, calculated the average scores for each scenario, as well as for each category. We performed a one-way ANOVA to test whether the uncertainty between subgroups was significantly different. 

#### 2.2.2. Scenario Selection Process

The average perceived uncertainty in each scenario is reported in Table 4. The scenario that was perceived to describe the least uncertain context was scenario 28 (*M* = 43.62) and the scenario perceived to describe the most uncertain context was scenario 5 (*M* = 62.02). These two scenarios were therefore selected for the next study phase. As the 30% decile was *d*_0,30_ = 49.03 and the 70% decile was *d*_0,70_ = 55.10, scenario 22 (*M* = 49.16) and 18 (*M* = 55.03) were selected to complete our four scenario set for the next study phase. This scenario choice matched our planned selection process while respecting our objective of selecting one scenario per subgroup. 

#### 2.2.3. One-Way ANOVA

We carried out a one-way ANOVA to investigate the effect of subgroups on the uncertainty perceived within the scenarios. We found a significant effect of subgroups on the perception of uncertainty in the scenarios, *F*(3, 595) = 68.20, *p* < 0.001. Scenarios in subgroup 4 (i.e., scenarios with four parameters in the “High uncertainty” modality, *M* = 58.53) were significantly perceived as describing a more uncertain context than those in subgroup 3 (i.e., scenarios with three parameters in the “High uncertainty” modality, *M* = 54.74), which in turn were perceived as describing a significantly more uncertain context than those in subgroup 2 (i.e., scenarios with two parameters in the “High uncertainty” modality, *M* = 49.03), which in turn were perceived as depicting a significantly more uncertain context than those in subgroup 1 (i.e., scenarios with a single parameter with one “High uncertainty” modality, *M* = 46.04). 

Then, we performed a one-way ANOVA to compare the effect of the scenario on the perceived uncertainty in the scenario for subgroup 1. There was no significant difference in perceived uncertainty in the scenario between at least two groups, *F*(4, 990) = 1.54, *p* = 0.190. Another one-way ANOVA with the same variables was performed for subgroup 2. It showed no significant difference in the perceived uncertainty in the scenario between at least two groups, *F*(9, 197) = 1.53, *p* = 0.132. We performed a third one-way ANOVA for subgroup 3 that did not show any significant difference in perceived uncertainty in the scenario between at least two groups, *F*(9, 195) = 1.00, *p* = 0.441. A fourth one-way ANOVA for subgroup 4 revealed once again no significant difference between at least two groups for perceived uncertainty in the scenario, *F*(4, 102) = 1.78, *p* = 0.130. Thus, these ANOVAs did not reveal any significant difference in the perception of uncertainty between the scenarios in the same subgroup.

In conclusion, our scenarios showed similar levels of perceived uncertainty in a given group, and different levels of perceived uncertainty compared to other groups. The perceived levels of uncertainty in scenarios were quite different and increased with the number of parameters in the “High uncertainty” condition.

## 3. Main Study

### 3.1. Method

#### 3.1.1. Population

For the main study, we also used a convenience sample recruited using the same procedure as the pretest. We recruited 1339 French-speaking individuals. We excluded 854 participants as they did not meet our inclusion criteria (i.e., participants needed to have a completion rate of at least 98%). Our final sample consisted of 485 individuals (*M_a_*_ge_ = 34.75, *ET*_age_ = 13.78, 294 women, 181 men, 10 others including 6 of which non-binary).

#### 3.1.2. Material

For this study, we used the four scenarios selected from the pretest and the two scenarios with the most extreme modalities for the five parameters that were presented as examples. For each of these six scenarios, participants first completed a block of questions about their vaccination intentions. Each participant first indicated whether they wanted to get vaccinated after reading the described sanitary condition using the question “Do you decide to get vaccinated?“ by answering either “Yes” or “No”. Then, participants indicated whether they would vaccinate their children by answering “Yes” or “No” to the question “Do you decide to have your child vaccinated?”. A third response option, “I don’t have children”, led to an additional question, “If you had any, would you decide to have your child vaccinated?” to which they could answer “Yes” or “No”. Finally, participants indicated whether they would recommend the vaccine by answering “Yes” or “No” to the question “Do you recommend the vaccine to others?”. The set of response options was deliberately written firmly and categorically to better match reality (e.g., putting a “Don’t know” option in a question asking whether participants would get vaccinated would have made no sense since in real life one gets vaccinated or not and not knowing whether one wants to be vaccinated means not getting vaccinated). We chose these measures because the vaccination of oneself, one’s children, and the discussions about vaccination and its potential recommendation are issues that arose during the COVID-19 pandemic and will usually arise in pandemic situations. They also appeared relevant as we thought the perceived level of uncertainty would modulate these items (e.g., the desire to protect one’s children may be exacerbated by the uncertainty of the situation, and one is generally reluctant to recommend treatment with uncertain effects). 

Next, participants answered a block of questions about their confidence in the decisions they had just made. At the beginning of this block, we asked participants “What do you think of the solution you have chosen to address the situation presented? Specifically, whether to vaccinate yourself, your children or to recommend the vaccine to your relatives”. Then, we presented to the participants the Confidence in Decision Quality subscale of the Attitudes toward Decision Process and Solution Questionnaire, developed by Aldag and Power and used by Jensen et al. [29]. This subscale includes the following three items: (1) I’m not sure my solution was appropriate, (2) My solution was a good one, and (3) I’m not confident about my solution. Participants indicated their agreement with these items on a 7-point Likert scale ranging from “Completely Disagree” (1) to “Completely Agree” (7). We chose to use a questionnaire because it provided a simple and quick-to-understand measure. A decisional gambling task such as the Iowa Gambling Task, often used for methodologies dealing with confidence in one’s decisions, would have made our methodology more cumbersome and we thought that a gambling task was too specific for our methodology. Among the questionnaires dealing with confidence in one’s judgment, temperature scales exist, but their translation into French is rather unsatisfactory (i.e., expressing one’s confidence in temperature by saying warm or hot is subtly difficult to translate into French). The Confidence in Decision Quality subscale seemed to be a good option because it was short, clear, and has already been used in a study on confidence in one’s judgments related to uncertain scenarios. 

Then, we presented to the participants the French version of the IUS-12 (i.e., the 12-item version of the Intolerance of Uncertainty Scale; [30]). This scale assesses both prospective IU (i.e., “propensity of individuals toward active information seeking as a way to reduce uncertainty/increase certainty”, [31]) and inhibitory IU (i.e., “an inhibition of actions or experience which is caused by uncertainty”, [31]). Response options were “Not at all characteristic of me” (1), “A little characteristic of me” (2), “Somewhat characteristic of me” (3), “Very characteristic of me” (4), and “Entirely characteristic of me” (5). 

Afterward, participants completed the Vaccination Attitudes Examination (VAX) validated by Martin and Petrie [32]. This 12-item scale measures general attitudes towards vaccines with items such as “I feel safe after being vaccinated” or “I worry about the unknown effects of vaccines in the future”. Participants responded on a scale ranging from “Strongly Disagree” (1) to “Strongly Agree” (6). 

Then, we presented the same socio-demographic block as in the pretest. 

#### 3.1.3. Procedure

The survey began with the same introductory page as in the pretest. Then, we showed the participants the set of scenarios selected after the pretest. For each scenario, participants were asked if they planned to get vaccinated and if they planned to vaccinate their children. If they did not have any, they were asked to answer by imagining what they would do if they did. Then they were asked to indicate whether they would recommend the vaccine. Finally, we measured their confidence in their decision, then their IU via the IUS-12, and their attitudes towards vaccines with the VAX. The questionnaire ended with socio-demographic questions (i.e., gender, age) and the same debriefing as in the pretest.

#### 3.1.4. Statistical Analysis

We investigated our three hypotheses by conducting three different types of analysis: (1) We conducted several repeated measures ANOVA to determine the effect of IU and attitudes towards vaccination on our dependent variables, (2) we then computed a difference in scores between the scenario describing the most uncertain situation and the one describing the least uncertain situation as an outcome. We conducted multiple linear regressions separately on each new outcome created, (3) and we performed an exploratory mediation analysis with IU as a predictor, attitudes towards vaccination as a mediator, and the difference in scores between the confidence in one’s decision of the most and least uncertain perceived scenario as a dependent variable.

### 3.2. Results

#### 3.2.1. Testing Whether a Higher Level of IU Leads to Lower Intentions to Get Vaccinated, to Have One’s Children Vaccinated, and to Recommend the Vaccine

**Figure 1:**
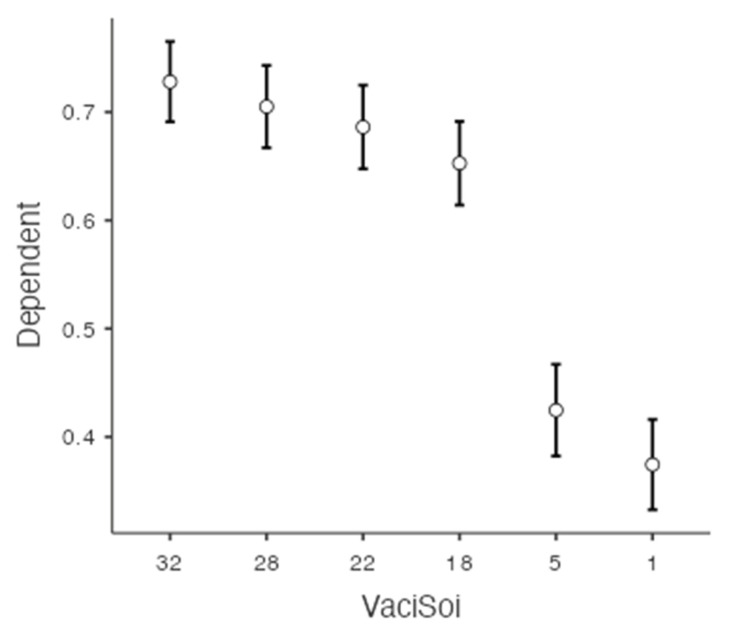
Scores for intention to get vaccinated according to the scenarios. Note: Dependent = Score for decision to get vaccinated; 32 = Scenario 32 (No modalities in “High uncertainty”); 28 = Scenario 28 (Mode of transmission: Remains unknown); 22 = Scenario 22 (Consequences: Whose long term consequences are unknown; and Effectiveness of the vaccine: We only have partial data on its effectiveness); 18 = Scenario 18 (Consequences: Whose long term consequences are unknown; Mode of transmission: Remains unknown; and Effectiveness of the vaccine: We only have partial data on its effectiveness); 5 = Scenario 5 (Knowledge about the virus: Recently discovered and not well known; Consequences: Whose long term consequences are unknown; Effectiveness of the vaccine: We only have partial data on its effectiveness; and Side effects: We do not yet know the risks of side effects); 1 = Scenario 1 (Knowledge about the virus: Recently discovered and not well known; Consequences: Whose long term consequences are unknown; Mode of transmission: Remains unknown; Effectiveness of the vaccine: We only have partial data on its effectiveness; and Side effects: We do not yet know the risks of side effects).

An omnibus repeated measures ANOVA did not show a significant effect of IU on the intention to get vaccinated, *F*(5, 238) = 1.42, *p* = 0.214, but it did show a marginally significant effect of attitudes towards vaccination on the decision to get vaccinated, *F*(5, 238) = 2.03, *p* = 0.072. Planned contrast showed a significant linear trend, *t*(484) = −15.80, *p* < 0.001, *d* = −0.72, quadratic trend, *t*(484) = −6.95, *p* < 0.001, *d* = −0.32, quartic trend, *t*(484) = 4.86, *p* < 0.001, *d* = 0.22, and a quintic trend, *t*(484) = 3.62, *p* < 0.001, *d* = 0.17, but no significant cubic trend, *t*(484) = 1.63, *p* = 0.104, *d* = 0.07. Scores for the intention to get vaccinated according to the conditions are shown in Figure 1. 

Another omnibus repeated measures ANOVA neither showed a significant effect of IU, *F*(5, 105) = 1.65, *p* = 0.145, nor a significant effect of attitudes towards vaccination, *F*(5, 105) = 1.17, *p* = 0.321, on the intention to get one’s children vaccinated. Planned contrast showed a significant linear trend, *t*(215) = −13.80, *p* < 0.001, *d* = −0.94, quadratic trend, *t*(215) = −6.86, *p* < 0.001, *d* = −0.47, quartic trend, *t*(215) = 4.74, *p* < 0.001, *d* = 0.32, and a quintic trend, *t*(215) = 3.10, *p* = 0.002, *d* = 0.21, but no significant cubic trend, *t*(215) = −0.04, *p* = 0.969, *d* < 0.002. Scores for the intention to get one’s children vaccinated according to the conditions followed the same graphical pattern as in Figure 1. 

A third omnibus repeated measures ANOVA did not show a significant effect of IU, *F*(5, 885) = 1.12, *p* = 0.345, but did show a significant effect of attitudes towards vaccination on the intention of having one’s child vaccinated if they had any, *F*(5, 885) = 2.82, *p* = 0.016. Planned contrast showed a significant linear trend, *t*(181) = −10.4, *p* < 0.001, *d* = −0.77, quadratic trend, *t*(181) = −4.63, *p* < 0.001, *d* = −0.34, cubic trend, *t*(181) = 2.68, *p* = 0.008, *d* = 0.20, quartic trend, *t*(181) = 3.25, *p* = 0.001, *d* = 0.24, and a quintic trend, *t*(181) = 2.21, *p* = 0.028, *d* = 0.16. Scores between conditions for this variable followed the same graphical pattern as in Figure 1. 

A final omnibus repeated measures ANOVA showed a marginally significant effect of IU, *F*(5, 238) = 1.95, *p* = 0.083, and a significant effect of attitudes towards vaccination on the intention to recommend vaccination in the described situation, *F*(5, 238) = 7.16, *p* < 0.001. Planned contrast showed a significant linear trend, *t*(484) = −17.70, *p* < 0.001, *d* = −0.80, quadratic trend, *t*(484) = −3.91, *p* < 0.001, *d* = −0.18, quartic trend, *t*(484) = 4.47, *p* < 0.001, *d* = 0.20, and a quintic trend, *t*(484) = 5.93, *p* < 0.001, *d* = 0.27, but no significant cubic trend, *t*(484) = 1.32, *p* = 0.188, *d* = 0.06. Scores for the intention to recommend vaccination according to the conditions followed the same graphical pattern as in Figure 1. Results are summarized in Table 5.

#### 3.2.2. Testing a Whether Higher Level of Negative Attitudes towards Vaccination Is Associated with Low Intentions to Get Vaccinated, to Have One’s Children Vaccinated, and to Recommend the Vaccine

We tested the first model including IU and attitudes towards vaccination which significantly predicted the difference in scores between the intention to get vaccinated of the most and least uncertain perceived scenario, *β* = −0.29, *t*(481) = −3.05, *p* = 0.002, *R*^2^ = 0.01. A second model including IU and attitudes towards vaccination significantly predicted the difference in scores between the intention to get one’s children vaccinated of the most and least uncertain perceived scenario, *β* = −1.36, *t*(212) = −4.45, *p* < 0.001, *R*^2^ = 0.01. A third model including IU and attitudes towards vaccination tended to predict the difference in scores between the intention of having one’s child vaccinated if they had any of the most and least uncertain perceived scenarios, *β* = −0.25, *t*(178) = −1.77, *p* = 0.078, *R*^2^ = 0.01. Lastly, a model including IU and attitudes towards vaccination significantly predicted the difference in scores between the intention to recommend vaccination in the described situation of the most and least uncertain perceived scenario, *β* = −0.41, *t*(481) = −4.30, *p* < 0.001, *R*^2^ = 0.02. 

#### 3.2.3. Testing a Whether High Level of IU Is Associated with Low Levels of Confidence in One’s Decision

As with H2, we tested H3 by computing the difference between the extreme scenarios. We tested a model including IU and attitudes towards vaccination which significantly predicted the difference in scores between the confidence in one’s decision of the most and least uncertain perceived scenario, *β* = 1.63, *t*(481) = 5.93, *p* < 0.001, *R*^2^ = 0.07. 

#### 3.2.4. Testing a Mediation Model of the IUS-12, VAX, and the Difference in Confidence in One’s Judgment Score

Results revealed a significant indirect effect of IU on the score difference of confidence in one’s decision, *β* = −0.10, *z* = −3.38, *p* < 0.001, but did not show a significant direct effect, *β* = 0.14, *z* = 1.59, *p* = 0.111. 

## 4. Discussion

### 4.1. Overall Results

#### 4.1.1. Aim of the Study

As the constant flow of information in our current society favors the increase in decision-making under uncertainty, related variables such as IU are increasingly studied. Recent research recognizes the negative effects of IU on problem-solving, especially in ambiguous situations [10,13,14], and points to the importance of studying the confidence in one’s own judgment in these contexts [16,17,18,19]. Whether or not to get vaccinated or get your children vaccinated during the COVID-19 pandemic is a great example of an uncertain situation in which individuals had to make a decision with significantly associated consequences. Inspired by this context, we conducted a study to investigate the impact of IU on the decision-making process under uncertainty (i.e., in a fictitious pandemic situation) and confidence in one’s decision, using a methodology with differing levels of perceived uncertainty. 

#### 4.1.2. Methodology Used

We conducted a pretest on 30 scenarios to accurately assess participants’ level of uncertainty perceived by an audience. We selected six scenarios with increasing levels of perceived uncertainty. In the core study, we tested the hypotheses that a high level of IU would be associated with low intentions to get vaccinated, to have their children vaccinated, and to recommend the vaccine (H1); that a high level of negative attitudes towards vaccination would be associated with low intentions to get vaccinated, to have their children vaccinated, and to recommend the vaccine (H2); and that a high level of IU would be associated with low levels of confidence in one’s decision (H3). Using a scenario-based methodology that presented situations with different levels of perceived uncertainty, we studied the impact of IU and attitudes towards vaccination on various variables that account for the vaccination decision (i.e., get vaccinated, vaccinate children, recommend vaccination).

#### 4.1.3. Pretest Results

Results of the pretest showed that the perceived level of uncertainty in the scenarios was homogeneous within a category and heterogeneous between categories, as we intended. The perceived level of uncertainty in the scenarios was distributed in an expected way, that is, an increasing number of parameters in the high uncertainty modality was associated with an increased level of perceived uncertainty. Despite few exceptions, the scenarios fell into the expected categories in that the scenarios judged to be the least uncertain were those with a single parameter in the “High uncertainty” modality, followed by scenarios with two parameters in this modality, then three modalities, then four, with slightly higher perceived uncertainty scores. However, beyond these results, we could not determine that one type of parameter was of greater importance than the others for the perception of uncertainty. It is interesting to note that in the pretest, the scenario judged to be the most uncertain was the one in which all parameters were in the “High uncertainty” modality, except for the “Transmission mode” parameter, and the scenario judged to be the least uncertain was the one in which none of the parameters were in the “High uncertainty” modality, except for the “Transmission mode” parameter. It can be speculated that this parameter has the least impact on the perception of uncertainty in such a situation. This is surprising, as it is an important element in viral dissemination that can potentially change the recommendations made [33]. People may not be aware of the importance of this variable and therefore do not adopt appropriate health behaviors.

#### 4.1.4. Main Phase Results

Regarding the main study, results showed a marginally significant effect of IU on the decision to recommend the vaccine. Attitudes towards vaccination had a marginally significant effect on the decision to get vaccinated and a significant effect on the decision to vaccinate one’s children, if they had any, and to recommend the vaccine. The planned contrasts revealed that in most cases there was a linear, quadratic, quartic, quintic, and sometimes cubic trend between IU, attitudes towards vaccination, and our measures. Linear regressions showed that IU and attitudes towards vaccination were significant predictors of the decision to get vaccinated, to vaccinate one’s children, to recommend the vaccine, and confidence in one’s decision. They were also marginal predictors of the decision to vaccinate one’s children if one had any. Finally, mediation analysis showed that IU has an indirect but not direct effect on the confidence in one’s decision through attitudes towards vaccination.

### 4.2. Understanding the Impact of IU and Attitudes towards Vaccination on Vaccination Decisions

Our ANOVA results suggest that there is no clear-cut differential impact of IU between the different scenarios on our measures, except perhaps on recommending vaccination. However, as IU is indeed a significant predictor of the majority of our measures, this implies that IU could predict vaccination decisions (i.e., getting vaccinated, having children vaccinated, and recommending the vaccine), regardless of the level of uncertainty perceived in the situation. This is in line with the results of Gillman et al. [4] who concluded in their study, “We found that individual trait-level differences in tolerance of uncertainty were associated with vaccine hesitancy”. A similar pattern was found for attitudes towards vaccination which predicted vaccination decisions, whereas our ANOVAs showed little evidence of differences based on the perceived level of uncertainty (except for recommendations and vaccinating one’s children if one had any). Furthermore, our planned contrasts revealed multiple significant patterns, among which, the linear shape was consistently the one with the highest effect size. We conclude that our data seem to show that as perceived uncertainty increases, individuals appear to have less intention to get vaccinated.

These results are very important as, if supported by replications in the future, they demonstrate that public health strategies need to be greatly reformed. Indeed, if vaccination decisions depend to a large extent on trait variables, then campaigns aimed at acting on environmental variables (e.g., trying to increase the level of certainty about the efficacy of a vaccine, increasing knowledge about the mode of transmission of disease) will have to be replaced by campaigns aimed at reducing the negative impact of trait variables (i.e., IU and attitudes towards vaccination, among others). This is crucial when analyzing the management of the COVID-19 pandemic in France, where the communication campaigns did not focus on these variables at all. Our results provide a more critical view of the measures that were taken during the COVID-19 pandemic, which, according to the importance of the factors we have highlighted, could probably be improved. Our results make it possible to refine future strategies without being subject to timing pressures, as was the case with COVID-19. We know of various approaches that help reduce IU in individuals, such as mindfulness, self-compassion, or Cognitive Behavioral Therapy (CBT) [34,35,36,37]. These highly individualized, mental health-focused methods will be difficult to mobilize collectively. A perspective for action would be to find strategies for the collective reduction in IU, partly based on these individualized practices. However, it seems important to focus information campaigns, which are a classic attitude-change vector used by public health authorities, on this type of method. Future research will need to determine more clearly how to disseminate methods that act on the traits identified in this study that might be effective.

### 4.3. Linking IU, Attitudes towards Vaccination, and Confidence in One’s Decision

The observed mediation is noteworthy in that it shows that confidence in one’s vaccination decision is influenced by attitudes toward vaccination, which, in turn, is influenced by IU. This demonstrates the importance of attitudes towards vaccination in the decision to get vaccinated and to recommend or have one’s relatives vaccinated, as previously demonstrated [28], while showing a link with two other variables of interest (IU and confidence in one’s decision). However, it should be noted that an individual’s decision made in a context in which they decide on the basis of given information should be distinguished from individuals’ attitudes towards vaccination and their level of IU, both of which are inherent to the individual and independent of the context. In addition, as our design was not experimental but cross-sectional, we cannot claim that we have demonstrated a causal chain. We conclude that, statistically, our data enable us to model a mediation pattern that will have to be experimentally confirmed in the future. The results of this exploratory mediation make sense if we consider IU as a construct closely related to stress and anxiety (i.e., this construct was originally studied in the context of anxiety disorders and has shown strong links with anxiety in studies about the perceived viral threat during a pandemic; [10,38]) and that stress can lead to favorable attitudes towards vaccination [39]. In addition, a recent study showed that IU was associated with more threat perception and pandemic-related stress, which were associated with more pandemic-related anxiety [4]. The links between IU and confidence in one’s judgment were also previously studied by Jensen et al. [29] who showed that a high level of IU can be associated with decreasing confidence in decisions across several blocks of decision-making, while a low level of IU is associated with increasing confidence. Though we wished to replicate these findings, our data led us to develop other hypotheses and explanations. However, this may be attributed to methodological differences (i.e., notably, to the pandemic content of our scenarios, which is a specific context with marked peculiarities, and which was not the subject of the scenarios used by Jensen et al. [29]). 

### 4.4. Limitations and Strength

Various limitations must be acknowledged to put the results of this study into perspective. Firstly, the choice of the experimental context (i.e., presenting a fictional pandemic) was an appropriate one insofar as it was topical and allowed for parallels to be drawn with the COVID-19 pandemic, as well as being a setting conducive to decision-making in an uncertain context. However, although the study began with a disclaimer stating that our study was not about the perception of COVID-19, we cannot be sure that the participants’ view of the COVID-19 pandemic did not affect their results. Indeed, COVID-19 has impacted society and our lives in many different ways, including the economy [40], education [40,41], relationships (notably couples and even gender-based violence [40,42]), physical health and lifestyle [40], mental health [40,43], and, unfortunately, infodemics and rumors [40]. As such, one can easily imagine that the pernicious effects of COVID-19 may be felt in people’s views of health crisis management and vaccination issues through the prism of those variables that were influenced during the pandemic. Furthermore, participants with strong views on vaccination issues (especially in the case of COVID-19) may have responded in a more extreme way than if the study had been conducted in a different health context or if we had studied decision-making for different topics. 

Secondly, several participants reported that they thought the study was a loop because each scenario was similar. Some participants may have dropped out of the study because they thought they were facing a computer bug as they did not notice the subtle differences between some scenarios. This limitation needs to be considered together with another one, that is, the number of participants excluded in the main study. Indeed, 854 participants out of 1339 were excluded, due to an insufficient completion rate of the study (less than 98%). We believe that the similarities between the scenarios may have discouraged many participants, who quit quickly or at least halfway through. However, it was necessary for all participants’ data to be complete to be used for our statistical analyses (i.e., to have expressed their views on all the scenarios) in order to make comparisons between the different scenarios, which justifies the exclusion of these 854 participants. Adding a disclaimer at the beginning of the study stating that participants would read quite similar but distinct scenarios could counter this limitation. 

Another limitation of our study is that we could have collected the reasons usually given by people with negative attitudes towards vaccination in order to include them in our regression models and neutralize their effects. Additionally, we could have refined the methodology in order to measure the reasons usually given by the anti-vaxxers to be able to statistically control for them. However, the VAX items mention the majority of reasons that anti-vaxxers generally provide to justify their attitudes. We can therefore consider that the VAX takes into account the elements that we did not specifically collect, and that this limitation does not jeopardize the meaning of our conclusions.

Finally, as with any study involving a convenience sample, we cannot be certain of the representativeness of our results for other populations and call for further studies to confirm our results.

However, there are also non-negligible strengths in our study. Firstly, the methodology of our study can be considered a strength in that our pretest ensured that we had accurate knowledge of the level of perceived uncertainty in each scenario, and the main phase presented scenarios of increasing levels of perceived uncertainty, which should have revealed an effect of the level of perceived uncertainty, if any. Future studies with a similar methodology will be needed to confirm this. The scenario library we created could be used for this purpose. Furthermore, the very creation of our scenarios is an important strength of our study. Indeed, our scenarios were designed to study the impact of several parameters of interest, so we rigorously measured their impact in the pretest. This allowed us to see in the main phase that the different levels of perceived uncertainty depending on the parameters displayed do not ultimately matter that much in the vaccinal decision, compared to the traits measured in the study. Furthermore, the richness of our vaccine decision measures (i.e., vaccination for self, children, and recommendation of the vaccine) provides us with important visibility on the behaviors that are actually impacted. We also note that our sample sizes are remarkable, both for the pretest and for the main phase of the study. This limits the possibility of our results being biased by a too small number of participants.

### 4.5. Futures Directions and Conclusions

One point to bear in mind from our study is the assessment of the perceived level of uncertainty in our scenarios during our pretest. We showed that, according to human intuition, an increasing number of parameters inspiring high uncertainty is associated with an increasing level of perceived uncertainty in a given situation. The main phase of the study also yielded very rich results as it showed the higher importance of trait variables (i.e., IU and attitudes towards vaccination) compared to an environmental variable (i.e., level of perceived uncertainty in a given situation) on the vaccination decision. More generally, this suggests that decisions may depend more on traits than on the environment, especially in uncertain situations and for high-stakes decisions.

However, our results should be compared with other studies that yielded mixed results regarding the impact of IU on the vaccinal decision (e.g., “We could not confirm the hypothesis that individuals who have a low tolerance to uncertainty will have increased coronavirus vaccine hesitancy/rejection”; “Past studies utilizing hypothetical scenarios have suggested that perceptions of uncertainty regarding vaccine safety may increase vaccine hesitancy”; [4,44]). Moreover, we note that the R^2^ of the regression analyses we performed is very low, which could indicate the minor importance of the variables we measured, and which leads to the need for future studies to confirm these results. One way of clarifying the results observed on these topics would be to consider different types of uncertainties, as suggested by Gillman et al. [4,45].

It will be necessary to study these constructs in the future to address tomorrow’s health issues. To further investigate and refine our findings, we could conduct a study with an experimental design presenting different scenarios with an increasing and controlled number of parameters to see the precise impact of each parameter on the vaccination decision. Indeed, considerable resources were invested during the COVID-19 pandemic to provide rational elements to individuals to help them decide to get vaccinated [39]. However, if we consider that the decision to get vaccinated is more dependent on traits, then this would appear to be useless and doomed because even if we reduce the level of perceived uncertainty, individuals will continue to make decisions according to their attitudes towards vaccination and their level of IU. A study design similar to our pretest, but with the main phase questions, would allow us to measure the impact of the nature of the information on which uncertainty varies on decisions and to adapt public health policies accordingly. This could be a determining factor in increasing adherence to vaccination policies and reducing the costs associated with communication on these issues.

## Figures and Tables

**Table 1:** Description of the five parameters modulating the uncertainty level elicited by the scenario through two uncertainty modalities.

Parameter	“Low Uncertainty” Modality	“High Uncertainty” Modality
Knowledge about the virus	That we have known and studied for many years	Recently discovered and not well known
Consequences	Which we know will not leave any after-effects	Whose long-term consequences are unknown
Mode of transmission	Is well known	Remains unknown
Effectiveness of the vaccine	Its effectiveness rate is known with certainty	We only have partial data on its effectiveness
Side effects	We are well aware of the possible side effects and their frequency	We do not yet know the risks of side effects

**Table 2:** Description of the content of the 30 scenarios presented in the pretest. Scenarios 1 and 32 were presented as references in the instructions.

Code Assigned to the Scenario	Content of the Scenario
2	C2PO is a very contagious virus that was recently discovered and is not well known. It mainly affects children and gives quite severe symptoms whose long-term consequences are unknown. The mode of transmission of the virus remains unknown. A vaccine has just been developed to fight against this pandemic which is getting worse. We only have partial data on its effectiveness and we are well aware of the possible side effects and their frequency.
3	C2PO is a very contagious virus that was recently discovered and is not well known. It mainly affects children and gives quite severe symptoms whose long-term consequences are unknown. The mode of transmission of the virus remains unknown. A vaccine has just been developed to fight against this pandemic which is getting worse. Its effectiveness is known with certainty and we do not yet know the risks of side effects.
4	C2PO is a very contagious virus that was recently discovered and is not well known. It mainly affects children and gives quite severe symptoms whose long-term consequences are unknown. The mode of transmission of the virus remains unknown. A vaccine has just been developed to fight against this pandemic which is getting worse. Its effectiveness is known with certainty and we are well aware of the possible side effects and their frequency.
5	C2PO is a very contagious virus that was recently discovered and is not well known. It mainly affects children and gives quite severe symptoms whose long-term consequences are unknown. The mode of transmission of the virus is well known. A vaccine has just been developed to fight against this pandemic which is getting worse. We only have partial data on its effectiveness and we do not yet know the risks of side effects.
6	C2PO is a very contagious virus that was recently discovered and is not well known. It mainly affects children and gives quite severe symptoms whose long-term consequences are unknown. The mode of transmission of the virus is well known. A vaccine has just been developed to fight against this pandemic which is getting worse. We only have partial data on its efficacy and we are well aware of the possible side effects and their frequency.
7	C2PO is a very contagious virus that was recently discovered and is not well known. It mainly affects children and gives quite severe symptoms whose long-term consequences are unknown. The mode of transmission of the virus is well known. A vaccine has just been developed to fight against this pandemic which is getting worse. Its effectiveness is known with certainty and we do not yet know the risks of side effects.
8	C2PO is a very contagious virus that was recently discovered and is not well known. It mainly affects children and gives quite severe symptoms whose long-term consequences are unknown. The mode of transmission of the virus is well known. A vaccine has just been developed to fight against this pandemic which is getting worse. Its effectiveness is known with certainty and we are well aware of the possible side effects and their frequency.
9	C2PO is a very contagious virus that was recently discovered and is not well known. It mainly affects children and gives quite severe symptoms but we know that they will not leave any after-effects. The mode of transmission of the virus remains unknown. A vaccine has just been developed to fight against this pandemic which is getting worse. We only have partial data on its effectiveness and we do not yet know the risks of side effects.
10	C2PO is a very contagious virus that was recently discovered and is not well known. It mainly affects children and gives quite severe symptoms but we know that they will not leave any after-effects. The mode of transmission of the virus remains unknown. A vaccine has just been developed to fight against this pandemic which is getting worse. We only have partial data on its efficacy and we are well aware of the possible side effects and their frequency.
11	C2PO is a very contagious virus that was recently discovered and is not well known. It mainly affects children and gives quite severe symptoms but we know that they will not leave any after-effects. The mode of transmission of the virus remains unknown. A vaccine has just been developed to fight against this pandemic which is getting worse. Its effectiveness is known with certainty and we do not yet know the risks of side effects.
12	C2PO is a very contagious virus that was recently discovered and is not well known. It mainly affects children and gives quite severe symptoms but we know that they will not leave any after-effects. The mode of transmission of the virus remains unknown. A vaccine has just been developed to fight against this pandemic which is getting worse. Its effectiveness is known with certainty and we are well aware of the possible side effects and their frequency.
13	C2PO is a very contagious virus that was recently discovered and is not well known. It mainly affects children and gives quite severe symptoms but we know that they will not leave any after-effects. The mode of transmission of the virus is well known. A vaccine has just been developed to fight against this pandemic which is getting worse. We only have partial data on its effectiveness and we do not yet know the risks of side effects.
14	C2PO is a very contagious virus that was recently discovered and is not well known. It mainly affects children and gives quite severe symptoms but we know that they will not leave any after-effects. The mode of transmission of the virus is well known. A vaccine has just been developed to fight against this pandemic which is getting worse. We only have partial data on its efficacy and we are well aware of the possible side effects and their frequency.
15	C2PO is a very contagious virus that was recently discovered and is not well known. It mainly affects children and gives quite severe symptoms but we know that they will not leave any after-effects. The mode of transmission of the virus is well known. A vaccine has just been developed to fight against this pandemic which is getting worse. Its effectiveness is known with certainty and we do not yet know the risks of side effects.
16	C2PO is a very contagious virus that was recently discovered and is not well known. It mainly affects children and gives quite severe symptoms but we know that they will not leave any after-effects. The mode of transmission of the virus is well known. A vaccine has just been developed to fight against this pandemic which is getting worse. Its effectiveness is known with certainty and we are well aware of the possible side effects and their frequency.
17	C2PO is a highly contagious virus that has been known and studied for many years. It mainly affects children and gives quite severe symptoms whose long-term consequences are unknown. The mode of transmission of the virus remains unknown. A vaccine has just been developed to fight against this pandemic which is getting worse. We only have partial data on its effectiveness and we do not yet know the risks of side effects.
18	C2PO is a highly contagious virus that has been known and studied for many years. It mainly affects children and gives quite severe symptoms whose long-term consequences are unknown. The mode of transmission of the virus remains unknown. A vaccine has just been developed to fight against this pandemic which is getting worse. We only have partial data on its effectiveness and we are well aware of the possible side effects and their frequency.
19	C2PO is a highly contagious virus that has been known and studied for many years. It mainly affects children and gives quite severe symptoms whose long-term consequences are unknown. The mode of transmission of the virus remains unknown. A vaccine has just been developed to fight against this pandemic which is getting worse. Its effectiveness is known with certainty and we do not yet know the risks of side effects.
20	C2PO is a highly contagious virus that has been known and studied for many years. It mainly affects children and gives quite severe symptoms whose long-term consequences are unknown. The mode of transmission of the virus remains unknown. A vaccine has just been developed to fight against this pandemic which is getting worse. Its effectiveness is known with certainty and we are well aware of the possible side effects and their frequency.
21	C2PO is a highly contagious virus that has been known and studied for many years. It mainly affects children and gives quite severe symptoms whose long-term consequences are unknown. The mode of transmission of the virus is well known. A vaccine has just been developed to fight against this pandemic which is getting worse. We only have partial data on its effectiveness and we do not yet know the risks of side effects.
22	C2PO is a highly contagious virus that has been known and studied for many years. It mainly affects children and gives quite severe symptoms whose long-term consequences are unknown. The mode of transmission of the virus is well known. A vaccine has just been developed to fight against this pandemic which is getting worse. We only have partial data on its efficacy and we are well aware of the possible side effects and their frequency.
23	C2PO is a highly contagious virus that has been known and studied for many years. It mainly affects children and gives quite severe symptoms whose long-term consequences are unknown. The mode of transmission of the virus is well known. A vaccine has just been developed to fight against this pandemic which is getting worse. Its effectiveness is known with certainty and we do not yet know the risks of side effects.
24	C2PO is a highly contagious virus that has been known and studied for many years. It mainly affects children and gives quite severe symptoms whose long-term consequences are unknown. The mode of transmission of the virus is well known. A vaccine has just been developed to fight against this pandemic which is getting worse. Its effectiveness is known with certainty and we are well aware of the possible side effects and their frequency.
25	C2PO is a highly contagious virus that has been known and studied for many years. It mainly affects children and gives quite severe symptoms but we know that they will not leave any after-effects. The mode of transmission of the virus remains unknown. A vaccine has just been developed to fight against this pandemic which is getting worse. We only have partial data on its effectiveness and we do not yet know the risks of side effects.
26	C2PO is a highly contagious virus that has been known and studied for many years. It mainly affects children and gives quite severe symptoms but we know that they will not leave any after-effects. The mode of transmission of the virus remains unknown. A vaccine has just been developed to fight against this pandemic which is getting worse. We only have partial data on its efficacy and we are well aware of the possible side effects and their frequency.
27	C2PO is a highly contagious virus that has been known and studied for many years. It mainly affects children and gives quite severe symptoms but we know that they will not leave any after-effects. The mode of transmission of the virus remains unknown. A vaccine has just been developed to fight against this pandemic which is getting worse. Its effectiveness is known with certainty and we do not yet know the risks of side effects.
28	C2PO is a highly contagious virus that has been known and studied for many years. It mainly affects children and gives quite severe symptoms but we know that they will not leave any after-effects. The mode of transmission of the virus remains unknown. A vaccine has just been developed to fight against this pandemic which is getting worse. Its effectiveness is known with certainty and we are well aware of the possible side effects and their frequency.
29	C2PO is a highly contagious virus that has been known and studied for many years. It mainly affects children and gives quite severe symptoms but we know that they will not leave any after-effects. The mode of transmission of the virus is well known. A vaccine has just been developed to fight against this pandemic which is getting worse. We only have partial data on its effectiveness and we do not yet know the risks of side effects.
30	C2PO is a highly contagious virus that has been known and studied for many years. It mainly affects children and gives quite severe symptoms but we know that they will not leave any after-effects. The mode of transmission of the virus is well known. A vaccine has just been developed to fight against this pandemic which is getting worse. We only have partial data on its efficacy and we are well aware of the possible side effects and their frequency.
31	C2PO is a highly contagious virus that has been known and studied for many years. It mainly affects children and gives quite severe symptoms but we know that they will not leave any after-effects. The mode of transmission of the virus is well known. A vaccine has just been developed to fight against this pandemic which is getting worse. Its effectiveness is known with certainty and we do not yet know the risks of side effects.

**Table 3:** The number of parameters in the “High uncertainty” modality per scenario.

	Number of Parameters in “High Uncertainty” Modality					
	0	1	2	3	4	5
Scenario code	32	31, 30, 28, 24, 16	29, 27, 26, 23, 22, 20, 15, 14, 12, 8	25, 21, 19, 18, 13, 11, 10, 7, 6, 4	17, 9, 5, 3, 2	1

**Table 4:** Average perceived uncertainty score in the scenario-by-scenario code and the number of parameters in the “High uncertainty” modality.

Number of Parameters in “High Uncertainty” Modality	Scenario Code	Average Perceived Uncertainty Score in the Scenario
1	28	43.62
1	24	44.25
1	16	45.58
2	8	46.80
2	20	46.83
1	31	47.38
2	15	47.71
2	27	48.17
2	12	48.74
2	22	49.16
1	30	49.26
2	26	49.57
2	14	50.06
2	23	50.26
3	19	52.09
3	25	52.64
2	29	53.21
3	13	54.11
3	4	54.45
3	7	54.51
3	18	55.03
3	11	55.26
3	6	55.54
4	9	55.89
3	21	56.77
3	10	57.08
4	3	57.37
4	17	57.90
4	2	59.77
4	5	62.02

**Table 5:** Summary of repeated measures ANOVAs results.

		Independent Variables	
		Intolerance of Uncertainty	Attitudes towards Vaccination
Measures	Getting Vaccinated	Not Significant	Marginally Significant
	Vaccinating one’s own children	Not Significant	Not Significant
	Vaccinate one’s own children if one had any	Not Significant	Significant
	Recommending the vaccine	Marginally Significant	Significant

## Data Availability

All the raw data and materials are available from the authors on request.
